# A 20-Minute Mindful Jazz Intervention Decreased Chronic Pain Patients’ Pain and Anxiety 4 Weeks Later: Results from a Pilot Randomized Controlled Clinical Trial

**DOI:** 10.3390/ejihpe15120239

**Published:** 2025-11-25

**Authors:** Sean D. Young, Adam Hanley

**Affiliations:** 1 Department of Emergency Medicine, University of California, Irvine, CA 92697, USA; 2 Department of Informatics, University of California, Irvine, CA 92697, USA; 3 College of Nursing, Florida State University, Tallahassee, FL 32306, USA; adam.hanley@fsu.edu

**Keywords:** chronic pain, mindfulness, music, intervention, randomized controlled trial

## Abstract

**Objectives:** Chronic musculoskeletal pain (CMP) is the leading cause of years lived with disability worldwide and the costliest health condition in the United States. Mindfulness is an effective treatment for CMP, but traditional mindfulness-based interventions (MBIs) are inaccessible for many CMP patients for both format and content reasons. An MBI that leverages music may be a more accessible approach. **Methods:** This pilot randomized controlled trial investigates the effects of a 20 min mindful jazz listening intervention with a 4-week self-directed practice period on chronic pain patients, comparing the daily mindful jazz listening (*n* = 27) with regular daily jazz listening (*n* = 30). We assess immediate and 4-week post-intervention outcomes for pain intensity, unpleasantness and anxiety. This study was conducted in accordance with ethical standards and is registered under the IRB (#3454) of the University of California, Irvine. **Results:** Results suggest that mindfully listening to jazz reduces pain and anxiety compared to the regular jazz group. **Conclusions:** Mindfully listening to jazz may be an effective, brief intervention for managing pain and anxiety in chronic pain patients, highlighting its potential as an accessible and engaging chronic pain management.

## 1. Introduction

Chronic musculoskeletal pain (CMP) stands as the foremost cause of years lived with disability across the globe and represents the most financially burdensome health issue in the United States (Dieleman et al., 2020; Moreno-Ligero et al., 2023; Sebbag et al., 2019; Vos et al., 2020). Mindfulness has proven to be an effective approach for managing CMP (Cardle et al., 2023; Piet et al., 2012), yet standard mindfulness-based interventions (MBIs) often remain out of reach for many individuals with CMP due to both their structure and content. Primarily, the conventional 8-week MBI model demands a significant commitment of time and resources, which may be unmanageable for a large portion of this population (Toivonen et al., 2020). Additionally, typical mindfulness exercises such as mindful breathing can be challenging or even uncomfortable for some CMP patients (Hanley et al., 2016; Lindahl et al., 2019; Toivonen et al., 2020). These obstacles to accessibility might be overcome by distilling the essential components of an effective 8-week MBI into a more concise format and by introducing a novel pain-relief strategy—mindful music listening—into this streamlined intervention, which may be more attractive to people living with CMP (Dvorak & Hernandez-Ruiz, 2021; Hernandez-Ruiz & Dvorak, 2021).

Meta-analyses already show that music, when used as a standalone intervention, can reduce pain (Lee, 2016). In line with this, recent research has found that individuals often favor having music as part of guided mindfulness sessions (Cheng et al., 2023; Dvorak & Hernandez-Ruiz, 2021; Hernandez-Ruiz & Dvorak, 2021). Importantly, music does not have to serve merely as a background; it can also become the central focus of mindfulness practice. Preliminary findings suggest that mindful music listening may offer benefits to people facing cancer (Lesiuk, 2016), recovering from stroke (Baylan et al., 2020), or experiencing depression (Eckhardt & Dinsmore, 2012). There has also been recent research suggesting that mindfully listening to music, especially improvisational jazz, may be helpful forfor pain management (S. D. Young et al., 2025). Given the widespread presence of music in everyday life, teaching those with CMP to use improvisational jazz as a mindfulness anchor could provide regular, accessible opportunities to engage in mindful pain management throughout the day.

Improvisational jazzmay be especially effective for a mindful music listening intervention due to its unfamiliarity and distinctive musical qualities. While most music interventions allow participants to select their preferred genre (Chlan & Heiderscheit, 2009), recent suggestions point to potential therapeutic advantages in mindfully listening to less familiar music, including jazz (Eckhardt & Dinsmore, 2012; S. D. Young et al., 2025). Unfamiliar music may naturally encourage present-moment attention, curiosity, and openness—key aspects of mindfulness—thereby enhancing the depth of mindful engagement during listening. Data on music preferences show that jazz is among the least purchased genres to Americans (Nielsen, 2014). The rapid and unexpected changes characteristic of improvisational jazz make each performance distinct and invite listeners to remain attentive to the unfolding present, much as the musicians themselves must do while improvising. Even though jazz “standards” may begin with a recognizable melody, each rendition is shaped by the musicians’ in-the-moment creativity, making every performance a unique, present-focused experience for both performers and attentive listeners.

Recent evidence supports the feasibility and effectiveness of mindful jazz listening for reducing pain and anxiety among individuals with chronic pain. In a pilot trial, S. D. Young et al. (2025) found that mindfully listening to jazz was a feasible and acceptable intervention and showed promise for reduced pain intensity and anxiety compared to control conditions, suggesting that music-based mindfulness approaches can meaningfully improve clinical symptoms in chronic pain populations (S. D. Young et al., 2025).

Informed by this preliminary work on improvisational jazz and our randomized controlled trials (RCTs) which reported the immediate and long-term benefits of brief (i.e., 15 to 20 min), single-session mindfulness interventions for pain (Garland et al., 2017; Hanley et al., 2021a, 2021b), we developed a 20 min, single-session mindful jazz listening intervention with a 4-week self-directed practice period. In this manuscript, we report on a two-arm (i.e., Mindful Jazz vs. Jazz Alone), randomized controlled clinical trial assessing the impact of mindful jazz listening on CMP patients’ immediate pain and anxiety, as well as their pain and anxiety during the first 4 weeks after intervention exposure. We hypothesized that, relative to the Jazz Alone condition, the Mindful Jazz condition would produce significantly larger decreases in pain intensity, pain unpleasantness, and anxiety over two different time scales: (1) from immediately before the intervention to immediately after, and (2) during the 4-week follow-up period as measured by daily pain and anxiety reports.

## 2. Materials and Methods

### 2.1. Participants

Study participants (*n* = 60) inclusion criteria were: self-reported 18 years of age or older, English-speaking American adults, living in the U.S., minimal exposure to jazz music, having a chronic musculoskeletal pain diagnosis with pain lasting at least 6 months and ≥4 on a 0 to 10 Numeric Rating Scale (NRS) during the week prior to study enrollment. Exclusion criteria included recent opioid use within the past 3 months; a current cancer diagnosis; significant neurological or psychiatric conditions, such as untreated post-traumatic stress disorder (PTSD) or bipolar disorder; engagement in other ongoing mindfulness or music-based interventions; or reported participation in clinical trials for chronic pain within the past year.

Consistent with current sample size recommendations for pilot studies, 30 participants were enrolled in each arm (i.e., Jazz Alone and Mindful Jazz). Following methods from prior studies using social media for recruitment, paid Facebook and Instagram advertisements emphasizing the study’s focus on chronic pain management on were used to recruit participants in the United States with chronic pain. Interested individuals were directed to complete a Qualtrics survey to help determine their eligibility. Next, eligible and interested individuals discussed study procedures with study personnel before reviewing and signing an electronic informed consent form. After providing informed consent, participants were randomly allocated to the Jazz Alone condition (control, *n* = 30) or the Mindful Jazz condition (treatment, *n* = 30) using a computer-generated randomization schedule created before study procedures began. We used paid advertisements based on prior research showing this is an effective recruitment tool in similar research contexts (Topolovec-Vranic & Natarajan, 2016), and then followed up with participants using a pre-screening survey, and additional follow-up by study personnel to verify eligibility and explain procedures.

### 2.2. Procedures

This was a pilot, randomized controlled clinical trial conducted online. All study procedures were approved by the University of California-Irvine Institutional Review Board (IRB) (#3454). The study was pre-registered (NCT05979012). In this manuscript, we only report on the immediate intervention effects and participants’ daily pain ratings. As waitlist control participants did not receive any intervention and did not provide daily pain ratings, they are not included in this manuscript.

We obtained written informed consent from all participants prior to study involvement. Participant interactions for training occurred via Zoom across the United States in both groups. A research assistant gave participants access to the Jazz Alone video and delivered the single-session Mindful Jazz intervention while watching the participants on Zoom to ensure they watched the video. The research assistant was not a professionally trained mental health or healthcare provider and had no formal experience as an interventionist other than as a staff researcher in prior trials.

The Jazz Alone intervention consisted of a video titled “What is Jazz”, which was developed by the National Museum of American History (Solidity, 2015). This video was 5 min long and described the history of jazz music and its broader influence on music and American culture. Participants learned about the improvisational nature of jazz. Participants in the Jazz Alone condition were not introduced to mindfulness or guided through a mindfulness practice.

The Mindful Jazz intervention was fully scripted and timestamped to facilitate appropriate pacing. First, participants were introduced to the intervention structure (1 min), pain neuroscience (1 min), and mindfulness (1 min). Next, participants were given an introduction to traditional mindfulness through a 3 min mindful breathing practice focused on the sensations of breathing, followed by 3 min of discussion about practice-related challenges. After teaching these basics of how and why mindfulness matters and is traditionally used, the mindful jazz listening practice instructions were introduced (1 min) and participants were guided to mindfully listen to the jazz song “Have You Met Miss Jones” by the Oscar Peterson Trio (4 min). During mindful jazz listening, participants were guided to notice and deconstruct the song (e.g., rhythm, melody, tempo) before noticing how mindfully listening to jazz impacted their pain levels and deconstructing any painful sensation remaining in the body (e.g., location, motion, density). This technique was adapted from Mindfulness-Oriented Recovery Enhancement (Garland, 2013). Discussion about the best parts of the mindful jazz listening practice and any practice-related challenges followed (2 min). Finally, the participant was assisted in developing a plan to practice mindfully listening to jazz (4 min).

**Figure 1:**
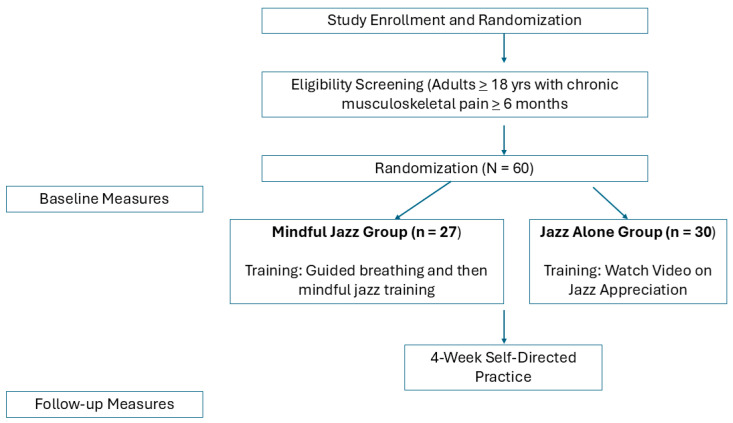
Participant Flow.

Participants in both conditions were instructed to listen to jazz music every day during a 4-week follow-up period (i.e., self-directed practice period). To help standardize participants’ jazz listening experience, participants were given a new jazz playlist each week during the follow-up period. The jazz playlist was the same for both conditions. The length of jazz listening was gradually increased over the 4 weeks: 10 min in week 1, 15 min in week 2, and 20 min in weeks 3 and 4. Towards the end of the study, participants in both groups were compensated for their time and effort with $50 Amazon gift cards provided after the completion of the study, which included baseline measures, the intervention session, and the 4-week follow-up period. This compensation was disclosed in recruitment materials and approved by the IRB. See Figure 1 for a flowchart of study methods.

### 2.3. Measures

Self-report measures of acute clinical symptoms and mindfulness-related states were administered immediately before and after intervention exposure. Participants were provided a personalized Qualtrics link, which allowed them to complete the measures privately. Pain intensity, pain unpleasantness and anxiety were measured with individual items rated on a 0–10 numeric rating scale (NRS) (Farrar et al., 2001; Garland et al., 2017; Hanley et al., 2021a; Lang et al., 2006; Q.-R. Young et al., 2015). During the 4-week follow-up period, participants were provided a daily Qualtrics link and asked to record their pain intensity, pain unpleasantness, and anxiety levels using the same NRS used before and after intervention exposure.

### 2.4. Sample Size Determination

The target sample size for this pilot study (i.e., *n* = 30 per arm) was informed by published recommendations (Teresi et al., 2022; Whitehead et al., 2016) and an audit of prior pilot and feasibility trials (Billingham et al., 2013).

### 2.5. Data Analyses

Analyses were thematically grouped to examine the primary variables of interest at two different time scales: (1) from immediately before to immediately after intervention exposure (i.e., immediate effects), and (2) during the 4-week follow-up period (i.e., follow-up effects). First, chi-squared tests and *t*-tests were used to investigate group equivalency at baseline. Then, generalized linear mixed modeling (GLMM) was used to analyze the immediate effects: pain intensity, pain unpleasantness, and anxiety. We used GLMM because it accommodates complex data structures, such as repeated measures, while accounting for both fixed and random effects. In each of these mixed models, outcome variables were regressed on condition (Jazz Alone vs. Mindful Jazz) and the respective baseline value in accordance with the classical analysis of covariance (ANCOVA) approach for analyzing clinical trial outcomes. The ANCOVA approach accounts for between-group differences at baseline, leading to more precise estimates of treatment effects (Frison & Pocock, 1992). A responder analysis was also conducted by calculating the proportion of participants with a ≥20% (i.e., minimally important) improvement in pain intensity from baseline (Smith et al., 2020). Finally, generalized linear mixed modeling was used to analyze the 4-week follow-up effects: pain intensity, pain unpleasantness, and anxiety. In each of these mixed models, the outcome variable was regressed on condition (Jazz Alone vs. Mindful Jazz), time (Day 0 through Day 28), and a condition X time interaction term. All tests and confidence intervals were two-sided and statistical significance was defined as a *p* value less than 0.05. All statistical analyses were conducted in SPSS version 29.

## 3. Results

### 3.1. Participant Demographics and Baseline Characteristics

Participants’ demographic information and baseline characteristics are reported in Table 1. All participants that began the Mindful Jazz intervention completed it. Three participants failed to complete the Jazz Alone intervention. As such, they were removed from statistical analyses. There were no statistically significant differences between the Jazz Alone and Mindful Jazz groups in demographics.

While none of the other variables of interest differed by group at baseline, a *t*-test indicated participants in the Jazz Alone condition reported significantly higher anxiety at baseline (Table 1). As such, the ANCOVA method was used to examine between-group differences in the immediate outcomes (Frison & Pocock, 1992; Lüdtke & Robitzsch, 2023).

### 3.2. Immediate Outcomes

Immediately after the intervention, generalized linear mixed modeling indicated participants in the Mindful Jazz condition reported significantly less pain intensity, pain unpleasantness and anxiety than participants in the Jazz Alone condition when accounting for the respective baseline values (Table 2). Examination of the unadjusted, post-intervention values indicated the Mindful Jazz condition ($\bar{x}$ = 4.70, SD = 1.86) decreased pain intensity by 1.47 points (i.e., compared to 0.67 points in the Jazz Alone condition ($\bar{x}$ = 6.00, SD = 2.02). Inspection of individual change scores revealed that more than half (16/30; 53%) of the Mindful Jazz participants reported a ≥20% reduction in pain intensity compared to only eight (30%) of the Jazz Alone participants.

### 3.3. Follow-Up Outcomes

During the 4-week follow-up period, participants completed an average of 16.58 symptom ratings (SD = 10.78) out of the 28 requested daily entries. There was no difference in the amount of follow-up data reported by condition (*t* = 0.42, *p* = 0.67). Generalized linear mixed modeling revealed no significant condition × time interactions, but significant main effects of condition were observed for each variable of interest. Participants in the Mindful Jazz condition reported significantly less pain intensity, pain unpleasantness, and anxiety during the 4-week follow-up period (Table 3). Significant main effects of time were observed for pain intensity (*F* = 13.52, *p* < 0.001) and pain unpleasantness (*F* = 8.39, *p* = 0.004), but not for anxiety (*F* = 4.06, *p* = 0.074).

## 4. Discussion

Results suggest that mindfully listening to jazz music can reduce pain and anxiety in individuals with chronic musculoskeletal conditions. CMP patients who received the Mindful Jazz intervention demonstrated immediate reductions in pain intensity and pain unpleasantness relative to participants in the Jazz Alone control condition. Specifically, the Mindful Jazz intervention decreased pain intensity by nearly 1.5 points (i.e., 23%) on a 0 to 10 numeric rating scale. The Mindful Jazz intervention produced an even larger decrease in pain unpleasantness: 2.2 points (i.e., 35%). These findings are consistent with results from single-session mindfulness interventions of a similar length targeting pain. In an RCT involving hospitalized patients reporting “intolerable pain” or “inadequate pain control” (*n* = 244), a 15 min mindfulness intervention immediately decreased pain intensity by 1.2 points (21%) and pain unpleasantness by 1.7 points (30%) (Garland et al., 2017). In three separate RCTs involving 15 to 20 min preoperative mindfulness interventions for knee or hip replacement patients (*n* = 285, *n* = 170, *n* = 118), immediate pain intensity decreases ranged from 1.1 to 2.1 points (28% to 50%) and immediate pain unpleasantness decreases ranged from 1.4 to 1.8 points (34% to 50%) (Hanley et al., 2021a, 2021b). Results from the present study extend these prior findings by suggesting the benefits of a non-traditional mindfulness intervention (i.e., mindfully listening to jazz) may be similar to those achieved by more traditional mindfulness interventions (i.e., mindful breathing). This finding has important practice implications as some individuals find traditional mindful breathing practices difficult or even uncomfortable (Hanley et al., 2016; Lindahl et al., 2019; Toivonen et al., 2020).

Critically, the pain relief observed in this study persisted across the 4-week follow-up period for participants in the Mindful Jazz condition. During the subsequent 4 weeks, pain intensity scores from the Mindful Jazz participants were nearly 1 point lower than those in the Jazz Alone condition. Mindful Jazz participants’ pain unpleasantness scores were mire than 1.25 points lower than their Jazz Alone counterparts. As such, it appears that a single-session, 20 min mindful jazz listening intervention with a 4-week self-directed practice period has the potential to help CMP patients feel better weeks later. These findings are consistent with accumulating evidence that single-session mindfulness interventions with practice follow-up can have a lasting impact on pain. In two RCTs involving total knee or hip replacement patients (*n* = 285, *n* = 118), 15 to 20 min, single-session mindfulness interventions delivered before surgery decreased patients’ pain and opioid use in the first 4 weeks after surgery and improved their physical function at the 6-week postoperative assessment (Hanley et al., 2021a, 2021b). Findings from the present study are also consistent with results from longer (i.e., 2 to 5 h), single-session behavioral interventions that have also been found to improve pain-related outcomes. (Darnall et al., 2021; Dindo et al., 2018; Ziadni et al., 2021). However, continuing to develop briefer interventions that seek to transform activities most people engage in every day (i.e., listening to music) into pain management tools has significant implications for treatment accessibility and (potentially) utilization.

We also found reductions in anxiety, a common chronic pain comorbidity (Beesdo et al., 2010; de Heer et al., 2014). Among CMP patients randomized to the Mindful Jazz intervention, anxiety immediately decreased by 1.1 points (19%) on a 0 to 10 numeric rating scale. This finding is consistent with results from earlier RCTs examining 15 min mindfulness interventions for hospitalized patients in pain as well as knee and hip replacement patients (Garland et al., 2017; Hanley et al., 2021a). Among hospitalized patients, mindfulness immediately decreased anxiety by 1.2 points (i.e., 30%) (Garland et al., 2017). In one RCT of knee and hip replacement patients, mindfulness immediately decreased preoperative anxiety by 1.9 points (i.e., 38%) (Hanley et al., 2021a). In another, mindfulness decreased anxiety by 1.8 points (i.e., 53%). Together, these studies suggest brief mindfulness interventions reliably decrease anxiety among pain patients. However, the present study is one of the first in which daily anxiety levels were assessed after the brief mindfulness intervention. During the 4-week follow-up period, anxiety scores from participants in the Mindful Jazz condition were over 1 point lower than the anxiety scores from participants in the Jazz Alone condition. As such, these early results suggest that a single-session mindful jazz listening intervention with a 4-week self-directed practice period may be able to decrease pain and anxiety at the same time.

This study raises several mechanistic questions that could be explored in future research. For example, it is already known that music interventions can reduce pain (Lee, 2016), but to what extent does mindful listening interact with or improve the effects of mindful listening. Although only limited research has studied the current topic (S. D. Young et al., 2025), improvisational music, such as jazz, might further reduce pain because of its novelty and potential attentional absorption—mechanisms that underline pain relief. Importantly, while both (mindful jazz and jazz alone) groups listened to jazz, the mindfulness component appeared to be related to the additional reductions in pain and anxiety, suggesting that mindful engagement with music may amplify its inherent benefits. In addition, incorporating music as a mindfulness anchor may further enhance accessibility and engagement, particularly for populations such as children and older adults. For example, mindful music listening can be tailored by adjusting factors such as familiarity, tempo, and lyrical content to match developmental or cognitive capacities, thereby potentially making mindfulness practice more approachable across diverse patient groups. Finally, it is unknown whether the current effects of the intervention might have resulted from an expectancy effect due to training participants that mindful listening will reduce their pain by leveraging prior research on the effects of mindfulness on pain.

Despite study strengths (e.g., randomized design, blinded assessment, longitudinal follow-up) and encouraging findings, limitations should also be noted. First, the study’s scope and impact are limited by its small sample size of 60 participants, which hinders the broader generalizability of these results. Future research should involve a larger sample of CMP patients in an RCT to more rigorously assess the effectiveness of mindful jazz listening. A second limitation is the absence of a mindful breathing (traditional mindfulness, without jazz) control condition. The absence of this condition precludes us from directly comparing the mindful jazz intervention with a traditional mindfulness intervention. Future studies may wish to incorporate multiple active control conditions (i.e., a traditional mindful breathing condition vs. a music listening without mindfulness training condition), in order to develop a more thorough understanding of the active therapeutic components in the mindful jazz intervention. Third, future studies should ensure their control conditions are time- and attention-matched. In the present study, participants in the Mindful Jazz condition received 20 min of mindfulness and jazz listening instruction from the research assistant while participants in the Jazz Alone condition were only provided a 5 min video, which they watched on their own. As such, it may be possible that time spent with the research assistant contributed to the observed between-group differences. Finally, the live delivery of the Mindful Jazz intervention versus the prerecorded video in the Jazz Alone condition may have introduced differential expectancy effects and time-with-facilitator bias that could be addressed in future studies.

## 5. Conclusions

This pilot study supports that mindfully listening to improvisational jazz may reduce CMP patients’ pain and anxiety compared to passive music listening alone. Critically, the observed reductions in pain and anxiety persisted across the 4-week follow-up period, suggesting that a brief, single-session, mindful jazz listening intervention with a 4-week self-directed practice period may have the potential to durably improve clinical symptoms. Larger clinical trials controlling for limitations are warranted to further assess mindful jazz listening as an accessible and engaging intervention for chronic pain management.

## Figures and Tables

**Table 1:** Demographic information of participants and average values for primary variables of interest at baseline by condition.

Baseline Demographic	Jazz Alone (*n* = 27)	Mindful Jazz (*n* = 30)	Test Statistic	*p*-Value *
	*n*	*n*		
Age, mean (SD)	44 (11.25)	40.77 (13.24)	*t* = 1.02	0.31
*Sex*, *n* (%)			Χ2 = 0.30	0.86
Female	22 (81.48%)	26 (86.67%)		
Male	5 (18.52%)	4 (13.33%)		
Ethnicity, *n* (%)			X2 = 0.91	0.63
Hispanic or Latino	4 (14.81%)	4 (13.33%)		
Not Hispanic or Latino	23 (85.19%)	25 (83.33%)		
Unknown	0 (0%)	1 (3.33%)		
Race, *n* (%)			X2 = 4.0	0.40
Asian	1 (3.70%)	1 (3.33%)		
Black or African American	2 (7.41%)	4 (13.33%)		
Native Hawaiian or Pacific Islander	1 (3.70%)	0 (0%)		
White	23 (85.19%)	24 (80.00%)		
Unknown	0 (0%)	1 (3.33%)		
Education, *n* (%)			X2 = 10.47	0.11
Bachelor’s degree	10 (37.04%)	12 (40.0%)		
Completed Graduate or Professional School	12 (44.44%)	12 (40.0%)		
Some College/Certificate	6 (22.22%)	4 (13.33%)		
Upper Secondary	1 (3.70%)	0		
Vocational/Trade School	3 (1.11%)	0		
Diploma or Equivalent (GED)	0	2 (6.64%)		
Variable	Jazz Alone	Mindful Jazz	Test Statistic	*p*-Value
Average Pain in the Week Prior to Study Enrollment	7.48 (1.19)	7.57 (1.43)	*t* = 0.24	0.81
Pain Intensity Immediately Before Intervention	6.67 (1.54)	6.17 (1.70)	*t* = 1.16	0.25
Pain Unpleasantness Immediately Before Intervention	6.78 (1.78)	6.27 (2.02)	*t* = 1.01	0.32
Anxiety Immediately Before Intervention	6.59 (1.80)	5.10 (3.32)	*t* = 2.14	0.04

Note: * Corresponds to *t*-test for continuous variables and chi-square test for association for categorical variables.

**Table 2:** Immediate outcomes by condition (adjusted analyses) *.

Variable	Jazz Alone	Mindful Jazz	*F*	*p*-Value
Pain Intensity	5.81 (5.20 to 6.42)	4.87 (4.29 to 5.45)	5.04	0.029
Pain Unpleasantness	5.55 (4.92 to 6.18)	4.27 (3.68 to 4.87)	8.56	0.005
Anxiety	4.43 (3.74 to 5.13)	3.11 (2.45 to 3.77)	7.44	0.009

Note: * Estimates are from linear mixed-models adjusting for baseline score.

**Table 3:** Main effects of condition over the 4-week follow-up period.

Variable	Jazz Alone	Mindful Jazz	*F*	*p*-Value
Pain Intensity	6.42 (5.88 to 6.95)	5.43 (4.91 to 5.95)	4.07	0.044
Pain Unpleasantness	6.11 (5.47 to 6.74)	5.08 (4.46 to 5.70)	4.10	0.043
Anxiety	5.95 (5.19 to 6.72)	4.84 (4.09 to 5.58)	3.98	0.046

## Data Availability

Data are available upon request, pending approval from the IRB.
